# Factors related to occupational accidents among nursing professionals from a public hospital in northern Brazil between the years 2009 to 2016

**DOI:** 10.5327/Z1679443520194387

**Published:** 2019-12-01

**Authors:** Glenda Keyla China Quemel, Letícia Caroline da Cruz Paula, Ivonete Vieira Pereira Peixoto, Osvaldo da Silva Peixoto, Jeferson Santos Araújo, Mônica Custódia do Couto Abreu Pamplona, Thayse Moraes de Moraes, Rodrigo Cesar Freitas da Silva, Bruno de Oliveira Santos

**Affiliations:** 1 Graduate Nursing Program, Universidade do Estado do Pará - Belém (PA), Brazil. Universidade do Estado do Pará Graduate Nursing Program Universidade do Estado do Pará Brazil; 2 Community Nursing Department, Universidade do Estado do Pará - Belém (PA), Brazil. Universidade do Estado do Pará Community Nursing Department Universidade do Estado do Pará Brazil; 3 Occupational Health Department, Faculdade Metropolitana da Amazônia - Belém (PA), Brazil. Faculdade Metropolitana da Amazônia Occupational Health Department Faculdade Metropolitana da Amazônia Brazil; 4 Graduate Nursing Program, Universidade Federal da Fronteira Sul - Chapecó (SC), Brazil. Universidade Federal da Fronteira Sul Graduate Nursing Program Universidade Federal da Fronteira Sul Brazil; 5 Graduate Nursing Program, Universidade do Estado do Pará - Belém (PA), Brazil. Universidade do Estado do Pará Graduate Nursing Program Universidade do Estado do Pará Brazil; 6 Statistical Modelling and Data Analysis Department, Universidade de Évora - Évora, Portugal. Universidade de Évora Statistical Modelling and Data Analysis Department Universidade de Évora Portugal; 7 Community Health Department, Universidade do Estado do Pará - Belém (PA), Brazil. Universidade do Estado do Pará Community Health Department Universidade do Estado do Pará Brazil

**Keywords:** accidents, occupational, nursing, occupational risks

## Abstract

**Background:**

Occupational accidents are considered public health problems, where there are gaps regarding studies on this theme in the northern region of Brazil with focus on nursing professionals.

**Aims:**

To analyze the factors related to occupational accidents in nursing professionals occurred in a large public hospital in Belém (PA) from January 2009 to December 2016.

**Method:**

Analytical, retrospective and longitudinal study with a quantitative approach by means of documentary census with a sample of 211 Work Accident Registry. We applied the Variance Analysis (ANOVA) tests for repeated measurements, chi-square of independence and t student for independent samples.

**Results:**

Female workers (72,04%), nursing technicians (88,15%) between the ages of 30 and 36 (34,13%), singles (45,18%) and public servants (95,74%) are most affected by occupational accidents, mainly typical accidents (91,94%). Sharps are the major causative agents (34,12%), morning (p=0,001) and afternoon (p=0,035) shifts have the highest mean number of accidents, and accidents occurred mostly in upper limbs (56,87%) and in the psychiatry sector (34,12%). The highest incidence rates of occupational accidents occurred in 2012 (49.5) and 2014 (60.2) per 1.000 professionals/year.

**Conclusion:**

As much as these results are similar to others evidenced and available in the literature, the study is particular because it is data from a public hospital in the northern region, an area that is not covered by studies of nature this.

## RESUMO |

**Introdução:**

Os acidentes de trabalho (AT) são considerados problemas de saúde pública, e existem lacunas quanto aos estudos sobre essa temática na Região Norte do Brasil com o foco em profissionais de enfermagem.

**Objetivo:**

Analisar os fatores relacionados aos AT entre a categoria profissionais de enfermagem em um hospital público de grande porte em Belém (PA), no período de janeiro de 2009 a dezembro de 2016.

**Métodos:**

Estudo analítico, retrospectivo e longitudinal com abordagem quantitativa, realizado por meio do censo documental com uma amostra de 211 registros de AT. Foram aplicados os testes estatísticos de análise de variância (ANOVA) para medidas repetidas, χ^2^ de independência e *t* de Student para amostras independentes.

**Resultados:**

Trabalhadores do sexo feminino (72,04%), técnicos de enfermagem (88,15%), entre 30 e 36 anos (34,13%), solteiros (45,18%) e servidores públicos (95,74%) são mais atingidos por AT, principalmente acidentes típicos (91,94%). Os perfurocortantes são os principais agentes causadores (34,12%), e os turnos da manhã (p=0,001) e da tarde (p=0,035) obtiveram maior média de número de acidentes. Os acidentes ocorreram majoritariamente em membros superiores (56,87%) e no setor de psiquiatria (34,12%). As maiores taxas de incidência de AT ocorreram nos anos de 2012 (49,5) e de 2014 (60,2) a cada 1.000 profissionais por ano.

**Conclusão:**

Por mais que esses resultados se assemelhem a outros evidenciados e disponíveis na literatura, particulariza-se o estudo por se tratar de dados provenientes de um hospital público da Região Norte, área de abrangência que não é contemplada por estudos dessa natureza.

## INTRODUCTION

Work accidents (WA) pose serious socioeconomic and public health problems^1^. The annual cost of WA in the formal labor market is about BRL 71 billion^2^. According to the International Labor Organization, WA and work-related diseases caused more than 2.3 million deaths in 2014, 14% corresponding to preventable WA^3^. In Japan, the largest number of WA in 2016 (n=1,718) occurred in the health and hygiene sector^4^.

In Brazil, the Administrative Ruling no. 1672/GM, from 2002, established the National Integrated Workers’ Health Care Network to implement health promotion, prevention, care and surveillance measures in all public health services. However, the reports of work-related incidents increased from 90,207 in 2010 to 157,333 to 2015, i.e. 74.40%. Along these 6 years, the largest number of reports (n=439,457, 54.30%) corresponded to severe WA, followed by incidents involving exposure to biological materials (n=276,669, 34.20%)^5^.

Health care workers are particularly susceptible to WA since they remain in direct contact with patients all along the working day. Factors such as competitiveness and mainly undervaluation of hospital work compel nursing professionals to have more than one job. This fact contributes to increase the risk of WA resulting from tiredness and exhaustion, in addition to poor working conditions^6^^,^^7^.

Studies performed in the Northeast^8^, South^7^, Southeast^9^ and Central-West^10^ regions of Brazil indicate that the main causes of WA among nursing professionals are related to improper disposal of sharps, needle recapping, inadequate medical waste containers, work overload, distraction and irritability. However, there is a gap in the literature as concerns the occurrence of WA among nursing professionals in public hospitals in the North region, i.e. our focus of interest. Indeed, the main innovative aspect of the present study is the possibility it brings to launch a substantial reflection on public hospitals in the North region of Brazil for the purpose of implementing prevention measures. The reason is that nursing professionals represent the largest occupational group in these institutions and consequently the most exposed to occupational hazards^11^^,^^12^. Detecting and preventing WA is increasing in relevance, therefore, the aim of the present study was to analyze factors related to WA among nursing professionals in a public hospital in Belém, Pará, Brazil, from January 2009 through December 2016.

## METHODS

The present longitudinal, retrospective, analytical and quantitative study was performed at a public hospital in Belém considered a referral facility for psychiatric, cardiologic, nephrological and high-risk pregnancy care. Data were collected by means of a document survey of 211 WA involving nursing professionals from January 2009 through December 2016. We considered reports of any kind of WA involving nursing professionals along the analyzed period. Records with missing, redundant or inconsistent information were excluded.

Data extracted from WA reports were entered in an ad hoc form that included the following variables: year, marital status, type of incident, occupational category (nurses or nursing technicians), sex (male or female), age, type of hazard (mechanical, biological or other), shift (morning, afternoon or night), involved body site (head/neck, lower limbs, upper limbs, chest/abdomen, polytrauma or other), location (surgery, clinical or psychiatry departments, intensive care unit-ICU, on the street, other) and causative agent (traffic accidents, body fluids, patients with a psychotic episode, sharps, uneven/slippery floor or other). For statistical analysis, variable body site was reduced to three categories (upper limbs, lower limbs and head/neck) since the frequency of all others was too low. The same was the case of variable causative agent; the frequency of chemicals and traffic accidents was very low and thus these agents were included in the category “other.”

We first entered the data in Microsoft Excel 2010 spreadsheets. Bivariate analysis was performed with software Statistical Package for the Social Sciences (SPSS) version 22. We performed descriptive analysis to characterize the sociodemographic profile of nursing professionals involved in WA. We used the two-tailed, Student’s t-test for independent samples to analyze the mean difference and variance of age relative to sex and occupational category. Next, we analyzed the mean difference and variance of WA according to occupational category. We estimated the mean number of WA according to type of hazard, shift, causative agent and location through repeated measures analysis of variance (ANOVA) and compared the variables which exhibited difference in means. The data met the normality assumption and thus we had resource to parametric tests. We analyzed the joint distribution and association between qualitative variables (shift and involved hazard) by means of the χ^2^ test of independence. In all the analyses the significance level was set to 5% (a=0.05) which ensures 95% of reliability to the results obtained.

The study was approved by the hospital research ethics committee, ruling no. 2,156,797. We complied with the privacy and confidentiality requirements described in the Resolution no. 466/2012 on research involving human beings. To ensure confidentiality, we did not record the workers’ names.

## RESULTS

The number of nursing professionals varied along the analyzed period, with an average of 709 per year. We analyzed 211 records of WA, which distribution according to sociodemographic variables is described in Table 1. About 72.04% (n=152) of the nursing professionals involved in WA were female and 88.15% (n=186) nursing technicians. The largest proportion of WA corresponded to workers aged 30 to 36 (n=72, 34.13%) and 37 to 43 (n=60, 28.44%) the youngest being 23 and the oldest 62, mean 39, standard deviation 8.81 years old. The largest proportion was of single employees, 42.18% (n=89). Most were civil servants (n=202, 95.74%) and were victims of typical accidents (n=194, 91.94%). About 46.92% (n=99) of incidents were associated to biological hazards and 34.12% (n=72) to mechanical hazards. About 44.55% (n=94) of incidents occurred in the morning and 34.60% (n=73) in the afternoon. About 56.87% (n=120) of accidents involved the upper limbs. Sharps and patients with a psychotic episode were the most frequent causative agents, 34.12% and 27.01% respectively. The largest proportion of incidents took place in the psychiatry department (n=72, 34.12%).

**Table 1.** Sociodemographic profile of nursing professionals involved in work accidents from January 2009 through December 2016, Belém, Pará, Brazil, 2009-2016 (n=211).

Variables	Absolute frequency (n)	Relative frequency
Sex		
Female	152	72.04%
Male	59	27.96%
Occupational category		
Nurses	25	11.85%
Nursing technicians	186	88.15%
Age (years)		
Up to 29	21	9.95%
30 to 36	72	34.13%
37 to 43	60	28.44%
44 to 50	29	13.74%
51 to 57	24	11.37%
Above 58	5	2.37%
Marital status		
Married	82	38.90%
Divorced	18	8.50%
Single	89	42.18%
Civil union	11	5.21%
Widowed	11	5.21%
Relationship employment		
Civil servant	202	95.74%
Private sector	8	3.79%
Other	1	0.47%
Type of accidents		
Typical	194	91.94%
Commuting	17	8.06%
Type of hazards		
Biological	99	46.92%
Mechanical	72	34.12%
Other	40	18.96%
Shift		
Morning	94	44.55%
Afternoon	73	34.60%
Night	44	20.85%
Involved body site		
Upper limbs	120	56.87%
Head and neck	44	20.85%
Chest/abdomen	13	6.16%
Lower limbs	21	9.95%
Polytrauma	12	5.69%
Other	1	0.48%
Causative agent		
Traffic accident	9	4.27%
Physical infrastructure	31	14.69%
Body fluids	25	11.85%
Patient with psychotic episode	57	27.01%
Sharps	72	34.12%
Chemicals	5	2.37%
Other	12	5.69%
Location		
Coronary care unit	5	2.37%
Obstetrics department	6	2.84%
Central sterile services department	8	3.79%
On the street	16	7.58%
Clinical departments	17	8.06%
Intensive care unit	23	10.90%
Surgery department	25	11.85%
Renal treatment department	23	10.90%
Psychiatry	72	34.13%
Other	16	7.58%

Table 2 describes the number of nursing professionals per year and the annual distribution of incidents. The highest rates of WA corresponded to 2012 (49.5/1000 workers/year) and 2014 (60.2/1000 workers/year). The incidence of WA exhibited a trend to decrease in the last 2 years.

**Table 2.** Annual number of nursing professionals, accidents and incidence, Belém, Pará, Brazil, 2009-2016 (n=211).

Year	Nursing staff	Accidents	Annual incidence (/1000 workers)
2009	597	23	38.5
2010	653	16	24.5
2011	680	25	36.7
2012	726	36	49.5
2013	732	26	35.5
2014	763	46	60.2
2015	756	25	33.0
2016	735	14	19.0

As shown in Table 3, Student’s t-test for independent samples did not find significant mean difference or variance in age between men and women or according to occupational category (nurses and nursing technicians). However, we found significant difference in age variance according to occupational category. The mean number of accidents exhibited significant difference between nurses and nursing technicians, but variance was not statistically significant. Variance was neither significant in regard to the number of accidents according to sex, but the mean number of incidents was.

**Table 3.** Statistical analysis of work accidents (Student’s t-test for independent samples), Belém, Pará, Brazil, 2009-2016 (n=211).

Occupational category					
	Nurses (n=25)		Nursing technicians (n=186)		Significance
	Mean	Standard deviation	Mean	Standard deviation	
Age	42.04	10.39	38.89	8.54	0.157
Number of accidents	2.50	1.51	13.29	6.47	0.000*
Sex					
	Male (n=59)		Female (n=152)		Significance
	Mean	Standard deviation	Mean	Standard deviation	
Age	39.94	8.88	37.51	8.46	0.072

*Statistically significant.

According to Table 4, repeated measures ANOVA detected significant difference in the mean number of incidents according to type of hazard, since it was larger for biological than for mechanical or other types of hazards. The mean number of accidents was significantly higher among workers allocated to the morning, followed by the afternoon shift. The highest mean number of accidents was related to sharps; this difference was statistically significant. Patients with a psychotic episode were the second leading causative agent, and the psychiatry department exhibited the highest mean difference. We did not find any significant difference in regard to the affected body site.

**Table 4.** Statistical analysis of work accidents (repeated measures ANOVA), Belém, Pará, Brazil, 2009-2016 (n=211).

Variables	df	Sum of squares	Mean square	F	Significance
Type of hazard	2	127.72	63.86	10.78	0.001*
Work shift	2	96.36	48.18	6.08	0.007*
Causative agent	4	78.64	19.66	3.65	0.027*
Location	5	62.97	12.59	11.88	0.000*
Body site	2	17.73	8.87	4.19	0.057

*Statistically significant; df: degrees of freedom.

The results of the χ^2^ test of independence, described in Table 5, indicate that variable work shift may influence the workers’ exposure to risk. In turn, we did not detect significant association for variables occupational category and type of hazard.

**Table 5.** Statistical analysis of work accidents (χ^2^ test of independence), Belém, Pará, Brazil, 2009-2016 (n=211).

Variables	χ^2^	p value
Occupational category × type of hazard	3.92	0.141
Work shift × type of hazard	9.59	0.048

## DISCUSSION

Most nursing professionals involved in WA were female, as shown in descriptive analysis (72.04%) and by the results of Student’s t-test. Similar findings are frequently reported in the literature, as for instance, in a study performed with nursing professionals at a hospital in Rio Grande do Sul, Brazil, in which most accident victims were female, 89.40%. The reason is that nursing is traditionally a predominantly female occupation^13^.

WA were most frequent among nursing technicians. This finding agrees with that reported in a study performed with 148 employees of a regional referral public hospital in a large city in Mato Grosso, Brazil, in which most accident victims were nursing technicians^10^. However, the results of the χ^2^ test indicate that variable occupational category had no relationship with type of hazard, since both nurses and nursing technicians were victims of accidents involving exposure to biological materials. These results call the attention to the need for standard prevention measures, continuous updating of the vaccination schedule and improvement of pre-employment and periodic medical examinations^14^.

According to several authors, younger workers are more susceptible to WA as a function of their lack of experience, insecurity and immaturity^15^^,^^16^. The causes of incidents might be related to lack of training, tiredness, having more than one job, substandard hospital infrastructure and organizational factors. In the present study, incidents were most frequent among the workers aged 30 to 36 (34.13%) which finding agrees with those reported in other studies^17^^,^^18^.

A survey of WA among Ministry of Health civil servants from 2010 to 2016 based on an analysis of WA reports found that 52.05% of incidents were commuting accidents^19^. Differently, in the present study typical accidents were the most frequent.

In regard to the type of involved hazard, our results agree with those reported in another study^20^, since the nursing staff is most exposed to biological hazards. Nevertheless, also mechanical hazards were relevant in our study, because the analyzed institution is a referral hospital for psychiatric care and many incidents are due to crashes, falls and violence in the psychiatry department. An integrative literature review evidenced that nursing professionals tend to underestimate the risks to which they are exposed and also to associate WA exclusively to biological hazards and occupational stress, although also other types of hazards should be taken into consideration, such as those of ergonomic, mechanical, physical and chemical nature^21^.

In regard to variable work shift, our results disagree from those of a study^22^ performed with 518 employees of the Cancer Hospital of Barretos, São Paulo, in which the largest proportion of incidents involved nursing professionals allocated to the night (35.00%), followed by the afternoon (33.30%) and the morning (31.70%) shift. An integrative review gathered scientific evidence on occupational risks and injuries among nursing professionals in the hospital environment, showing that accidents happen more in the morning shift^23^. Therefore, the literature diverges regarding the shift with the highest number of accidents, probably due to the workplace characteristics. Differently, we found that frequency was highest in the morning and afternoon, perhaps because the flow of patients is higher, as also is the rate of procedures likely to cause WA.

Most incidents involved the upper limbs (56.87%). Other authors reported the hands as the most prevalent involved body site, which is expectable, since the hands are the main working tool for the nursing staff^24^^,^^25^^,^^26^. However, ANOVA failed to detect significant difference among the reported body sites. This finding indicates that personal protective equipment (PPE) for all body parts should be worn during all types of procedures, from the most simple to the most complex.

A study performed in the Federal District, Brazil, found that sharps injuries were the most prevalent type of incidents, 72.20%, mainly involving non-hollow needles.^24^ Another study performed in São Paulo reported similar results, since 91.60% of incidents with exposure to biological hazards were sharps injuries in association with needle recapping. These findings agree with those of the present study.

However, we should call the attention to the vulnerability of the nursing staff in psychiatric hospitals, since according to several authors^27^^,^^28^ psychotic patients may see procedures such as mechanical, physical or verbal restraints and administration of medication, among others, as threats and thus react violently. In a study performed with nursing technicians at two public psychiatric hospitals in Belo Horizonte, Minas Gerais, Brazil, 76.8% of the participants reported to have been exposed to physical violence in the workplace.

We could not find any study that analyzed the association between WA and marital status.

Most accidents involved civil servants, which was expected because the analyzed institution is a public hospital.

The incidence of WA (per 1000 workers/year) was highest in 2012 and 2014. While we could not find any data in the literature to account for this higher incidence in 2012, a study based on data available from the System of Information of Notifiable Diseases to describe WA involving exposure to biological materials from 2010 through 2016 found that the highest rate nationwide corresponded to 2014 (16.84/workers/year) while in Pará to 2014 and 2016^29^.

The limitations of the present study derive from the legibility of records and the excessive bureaucracy that hinders the access to data. These aspects call the attention to the need to raise awareness on the relevance of properly filled reports forms, since lack of compliance interferes with the interpretation of results, as well as with the implementation of actions for workplace improvement. Similarly, we suggest simplifying the procedure to access data to thus contribute to foster scientific research.

## CONCLUSION

Most incidents were typical accidents and involved women, nursing technicians, workers aged 30 to 36, single and civil servants. Biological and mechanical hazards were the most frequently associated with WA, and sharps were the most common causative agent. Prevalence of WA was highest among workers allocated to the morning shift and the psychiatry department and involved the upper limbs. We call the attention to the need to strengthen preventive measures and organizational policies to reduce the rate of WA among nurses and nursing technicians, including availability of PPE for all types of procedures.

Although our results are similar to others reported in the literature, they are relevant because they correspond to a public hospital in the North region of Brazil, which has not been addressed in previous studies. Therefore, they represent a contribution to the strengthening of public policies and improvement of the care provided to the targeted population of workers. While we focused on the nursing staff, additional studies are needed to minimize WA among all occupational groups.

## References

[1] Medina FS, Maia MZB (2016). A subnotificação de LER/DORT sob a ótica de profissionais de saúde de Palmas, Tocantins. Revista Brasileira de Saúde Ocupacional.

[2] Malta DC, Stopa SR, Silva MMA, Szwarcwald CL, Franco MS, Santos FV (2017). Acidentes de trabalho autorreferidos pela população adulta brasileira, segundo dados da Pesquisa Nacional de Saúde. Ciênc Saúde Coletiva.

[3] International Labour Organization (2015). Global Trends on Occupational Accidents and Diseases.

[4] Associação Japonesa de Segurança e Saúde Industrial (2017). Guia Geral de Saúde Industrial 2017.

[5] Brasil. Ministério da Saúde (2017). Boletim Epidemiológico. Vigilância em Saúde do Trabalhador: um breve panorama.

[6] Santos PHS, Reis LA (2016). Underreporting of Accidents at Work in Nursing Professionals: Integrative Review. J Nurs UFPE.

[7] Ceron MDS, Magnago TSBS, Camponogara S, Luz EMF, Beltrame MT, Bottino LD (2015). Prevalência e fatores associados aos acidentes de trabalho no serviço hospitalar de limpeza. Rev Pesq Cuidado Fundamental Online.

[8] Santos SR, Novaes CO (2018). Profile of accidents with sharps among health professionals from a hospital of the public network at São Luís city. Rev Fun Care Online.

[9] Cedraz RO, Gallasch CH, Pérez EF, Gomes HF, Rocha RG, Mininel VA (2018). Gerenciamento de riscos em ambiente hospitalar: incidência e fatores de riscos associados à queda e lesão por pressão em unidade clínica. Esc Anna Nery.

[10] Carvalho DC, Rocha JC, Gimenes MCA, Santos EC, Valim MD (2018). Acidentes de trabalho com material biológico na equipe de enfermagem de um hospital do Centro-Oeste brasileiro. Esc Anna Nery.

[11] Rezende LCM, Leite KNS, Santos SR, Monteiro LC, Costa MBS, Santos FX (2015). Occupational accidents and their impact to the health of nursing professionals. Rev Baiana Enferm.

[12] Dos Santos NC, Santos LS, Camelier FWR, Maciel RRBT, Portella DDA (2017). Tecnologias aplicadas à promoção da saúde do trabalhador: uma revisão sistemática. Rev Bras Med Trab.

[13] Silva RM, Zeitoune RCG, Beck CLC, Souza SBC, Santos E (2015). Chronotype and work accidents in the nursing team of a surgical clinic. Texto Contexto Enferm.

[14] Corrêa LBD, Gomes SCS, Ferreira TF, Caldas AJM (2017). Fatores associados ao uso de equipamentos de proteção individual por profissionais de saúde acidentados com material biológico no Estado do Maranhão. Rev Bras Med Trab.

[15] Alves AP, Pedrosa LAK, Coimbra MAR, Miranzi MAS, Hass VJ (2015). Prevalência de transtornos mentais comuns entre profissionais de saúde. Rev Enferm UERJ.

[16] Faria NMX, Klosinski RFS, Rustick G, Oliveira LM (2018). Saúde mental dos trabalhadores da saúde pública em Bento Gonçalves, no Rio Grande do Sul. Rev Bras Med Trab.

[17] Negrinho NBS, Malaguti-Toffano SE, Reis RK, Pereira FMV, Gir E (2017). Fatores associados à exposição ocupacional com material biológico entre profissionais de enfermagem. Rev Bras Enferm.

[18] Cardoso MLLO, Slob EMG (2015). Enfermagem: características dos profissionais que sofrem acidentes com material biológico. Rev Recien.

[19] Brasil. Ministério da Saúde (2018). Levantamento de registros de acidente em serviço de servidores do ministério da saúde em Brasília/DF nos anos de 2010 a 2016.

[20] Garbaccio JL, Regis WCB, Silva RMC, Estevão WG (2015). Acidentes Ocupacionais com a equipe de enfermagem da atenção hospitalar Cogirate Enferm.

[21] Silva RSS, Madeira MZA, Fernandes MA, Batista OMA, Brito BAM, Carvalho NAR (2017). Riscos ocupacionais entre trabalhadores de enfermagem em Unidade de Terapia Intensiva. Rev Bras Med Trab.

[22] Luize PB, Canini SRMS, Gir E, Malaguiti-Toffano SE (2015). Conduta após exposição ocupacional a material biológico em um hospital especializado em oncologia. Texto Contexto Enferm.

[23] Bezerra AMF, Bezerra KKS, Bezerra WKT, Athayde ACR, Vieira AL (2015). Riscos ocupacionais e acidentes de trabalho em profissionais de enfermagem no ambiente de trabalho. Rev Bras Educ Saúde.

[24] Santos SVM, Macedo FRM, Silva LA, Resck ZMR, Nogueira DA, Terra FS (2017). Acidente de trabalho e autoestima de profissionais de enfermagem em ambientes hospitalares. Rev Latino-Am Enfermagem.

[25] Rodrigues PS, Sousa AFL, Magro MCS, Andrade D, Hermann PRS (2017). Acidente ocupacional entre profissionais de enfermagem atuantes em setores críticos de um pronto-socorro. Esc Anna Nery.

[26] Donatelli S, Vilela RAG, Almeida IM, Lopes MGR (2015). Acidente com material biológico: uma abordagem a partir da análise das atividades de trabalho. Saúde Soc.

[27] Paula GS, Oliveira EB, Silva AV, Souza SRC, Fabri JMG, Guerra AO (2017). Violência relacionada ao trabalho na psiquiatria: percepção dos trabalhadores de enfermagem. Rev Eletrônica Saúde Mental Álcool Drog.

[28] Vieira GLC (2017). Agressão física contra técnicos de enfermagem em hospitais psiquiátricos. Rev Bras Saúde Ocup.

[29] Gomes SCS, Caldas ATM (2019). Incidência de acidentes de trabalho com exposição a material biológico em profissionais de saúde no Brasil, 2010-2016. Rev Bras Med Trab.

